# Report of a rare case of histiocytic necrotizing lymphadenitis with bilateral pleural effusion diagnosed via cervical lymph node biopsy

**DOI:** 10.1590/1516-3180.2016.0333170217

**Published:** 2017-07-31

**Authors:** Xuchun Liu, Shubin Huang, Guohua Jiang

**Affiliations:** I MD. Specialist, Department of Respiratory Medicine, Chizhou People’s Hospital, Chizhou, Anhui, China.; II MD. Specialist, Department of Pathology, Chizhou People’s Hospital, Chizhou, Anhui, China.; III MD, PhD. Professor, Department of Respiratory Medicine, Chizhou People’s Hospital, Chizhou, Anhui, China.

**Keywords:** Histiocytic necrotizing lymphadenitis, Pleural effusion, Tuberculosis, Lymph nodes, Biopsy

## Abstract

**CONTEXT:**

Histiocytic necrotizing lymphadenitis (HNL) is a rare disorder that is often benign and self-limiting. There have been reports of co-occurrence of HNL with other diseases, including systemic lupus erythematosus, hemophagocytic syndrome and antiphospholipid syndrome.

**CASE REPORT:**

Here, we report a case in which a patient experienced unexplained fever, swelling of the cervical lymph node and bilateral pleural effusion and was ultimately diagnosed with HNL based on results from a lymph node biopsy. After treatment with glucocorticoid, the patient regained normal body temperature, the swelling of the lymph nodes disappeared and the pleural effusion was reabsorbed.

**CONCLUSIONS:**

The pathogenesis of HNL remains unclear, and pleural effusion is rarely reported in HNL patients. We presented this case to improve diagnostic awareness of this condition among clinicians and help reduce the likelihood of misdiagnosis.

## INTRODUCTION

Histiocytic necrotizing lymphadenitis (HNL) is a rare disorder, and its pathogenesis remains unclear. Some scholars believe that HNL is not an independent disease and that it may be a manifestation of an underlying autoimmune disorder; as such, the diagnosis and treatment for HNL should be targeted to the underlying disease instead. There have been reports of co-occurrence of HNL with other diseases, including systemic lupus erythematosus (SLE), hemophagocytic syndrome and antiphospholipid syndrome.^1^^,^^2^

However, pleural effusion has rarely been reported in HNL patients. Here, we report a case in which the patient experienced unexplained fever, swelling of the cervical lymph node and bilateral pleural effusion and was ultimately diagnosed with HNL based on the results from a lymph node biopsy.

## CASE REPORT

A 26-year-old woman was hospitalized due to anorexia and fatigue that had lasted one month. Ten days before admission, the patient began to experience intermittent chills and a fever between 38.5 and 40 °C. Physical examination on admission revealed that both cervical lymph nodes were swollen and tender, more obviously so on the left side. Double lung auscultation detected lower breathing sounds.

Laboratory examinations revealed the following: white blood cell count, 2.45 × 10^9^/l; neutrophils: 48.70%; lymphocytes: 42%; red blood cell count: 3.9 × 10^12^/l; hemoglobin: 101 g/l; platelets: 141 × 10^9^/l; erythrocyte sedimentation rate: 30 mm/h; procalcitonin: 0.095 ng/ml; and C-reactive protein: 19.11 mg/l. The results for tumor markers, autoimmune antibodies, anti-neutrophil cytoplasmic antibodies and blood cultures were normal. The T-SPOT and purified protein derivative tests for tuberculosis were negative, as were tests for pleural tuberculosis antibodies. Bone marrow cytological analysis revealed bone marrow hyperplasia.

**Figure 1.**
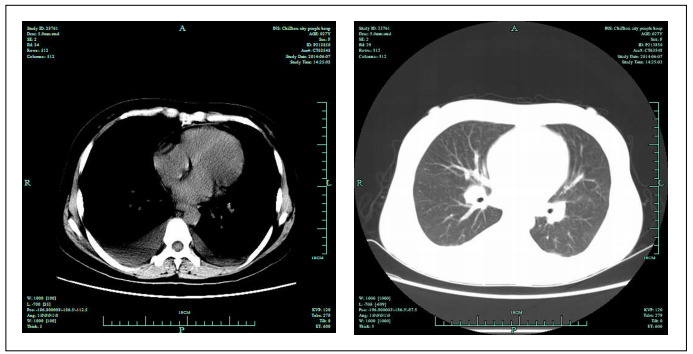
Computed tomography of the chest, revealing slight swelling of the mediastinal lymph nodes and a small amount of bilateral pleural effusion, with slightly more on the right side than on the left.

Computed tomography (CT) of the chest revealed slight swelling of the mediastinal lymph nodes and a small amount of bilateral pleural effusion, which was slightly more severe on the right side (Figure 1). Ultrasonography of the neck revealed multiple swollen bilateral supraclavicular lymph nodes, with some as large as 16 × 7 mm. Ultrasonography of the upper abdomen and uterus did not reveal any anomaly.

The pleural puncture technique was applied to drain excessive fluid, and Rivalta test results were positive. The pleural effusion pH was 7.35 (normal, 6.8-7.6), proteins were 32 g/l (normal, 0-30), and lactate dehydrogenase, adenosine deaminase and carcinoembryonic antigen levels were 2186 U/l (normal, 0-200), 30 U/l (normal, 0-40) and 1.64 ng/ml (normal, 0-6.5), respectively. The total number of nucleated cells in the pleural effusion was 3886 × 10^6^/l (normal, 100-500 × 10^6^/l). Repeated pleural fluid cytological analysis revealed a large quantity of lymphocytes. Pleural fluid cultures were negative.

The patient underwent two weeks of anti-inflammatory treatment with 0.2% levofloxacin, 300 ml daily administered by means of intravenous drip, which was ineffective. She still experienced intermittent fever, and ultrasonography revealed continued pleural effusion.

**Figure 2.**
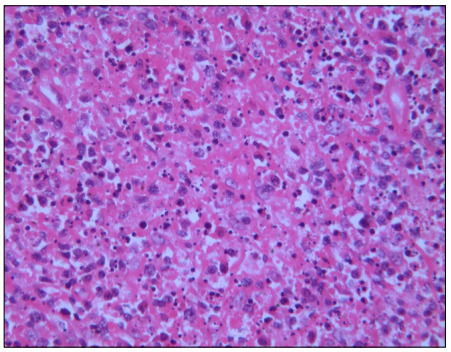
Biopsy specimen from the left cervical lymph node. Pathological manifestation showing fibrin deposition, a large amount of nuclear debris and large mononuclear cell aggregates within the necrotic regions (hematoxylin and eosin staining; magnification × 400).

A biopsy on the left cervical lymph node showed scattered fibrin deposition, a large amount of nuclear debris and large mononuclear cell aggregates in the necrotic area (Figure 2). The pathological diagnosis was histiocytic necrotizing lymphadenitis.

**Figure 3.**
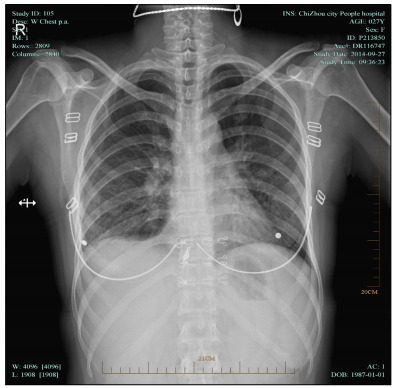
Chest radiograph, showing that the double lung texture disorder and bilateral pleural effusion had disappeared.

Antibiotics were discontinued after two weeks of treatment with intravenous infusion of levofloxacin, and 10 mg of oral prednisone was administered three times daily. Within three days, the patient’s body temperature gradually returned to normal. After general improvement during a one-week observation period, she was discharged and was prescribed a reduced dose of oral prednisone. Three months later, a chest radiograph showed that the double lung texture disorder and bilateral pleural effusion had disappeared (Figure 3).

## DISCUSSION

Histiocytic necrotizing lymphadenitis is a rare inflammatory disease of the lymph nodes. It is more common in young women and has a natural course of 1-4 months,^3^ but relapse may occur. The cause of HNL remains unclear, but it is probably associated with infections and autoimmune disorders. A variety of viruses, such as adenovirus, Epstein-Barr virus and herpes virus, are reportedly associated with the disease. HNL onset is commonly associated with acute or subacute clinical manifestations, such as swollen lymph nodes, fever and neutropenia. The most commonly affected lymph nodes are cervical, followed by axillary. Intermittent fever, fatigue, joint pain, hepatosplenomegaly and skin rashes are also common symptoms. Rarely, HNL patients exhibit complex clinical manifestations involving multiple organ systems, similar to those for rheumatic diseases.

It has been reported in the literature that the clinical manifestations of HNL are similar to those of certain connective tissue diseases, such as adult-onset Still’s disease and SLE. There are also reports of HNL co-occurring with these diseases, with the strongest link to SLE. Reports have suggested that HNL can occur before, after or concurrently with SLE pathogenesis.^4^ In particular, anti-nuclear antibody-positive HNL is more likely to develop into SLE, and certain scholars even hold that HNL is a unique manifestation of SLE.^5^ Additionally, certain clinical manifestations of lymphoma are very similar to those of HNL; as such, HNL patients are often misdiagnosed as having malignant lymphoma.

Noninvasive diagnostic methods are limited in their effectiveness. At present, application of 18F-deoxyglucose (18F-FDG) positron emission tomography (PET)/CT is widely used as a tumor metabolism imaging technique for determining lymph node malignancy. However, studies using FDG imaging and PET/CT have suggested that in HNL patients, lymph node uptake values ranged from 2.05 to 13.94, with an average of 6.25 ± 3.32. Cervical lymph nodes often have a higher uptake value. Evidently, PET/CT scans alone are not sufficient to differentiate between HNL and lymphoma, but the biopsy site can be determined using the standard uptake value.^6^

Pathological and immunohistochemical examinations are important tools for differential diagnosis. In 1995, Kuo^7^ further defined HNL into three subtypes: proliferative, necrotic and xanthoma-like, which may reflect the different stages of disease development, namely hyperplasia-necrosis-xanthoma or granulation-reabsorption-recovery. The pathological characteristics of HNL include a patchy lymph node disorder, commonly found in the juxtacortical area with visible fibrinoid necrosis and a large amount of nuclear debris. Necrotic areas are surrounded by proliferative cells, but granulocytes and plasma cells are rare or absent. Specialized or tufted CD68-positive and MPO-positive cells are important immunohistochemical features of HNL.^8^ T-cells are the most commonly found lymphocytes, while B-cells and natural killer cells are rarer. Using an electron microscope, a large number of apoptotic bodies surrounded by mononuclear histiocytes and scattered T-cells undergoing apoptosis were found in the lymph nodes of HNL patients. These three observations are closely related, and they suggest that T-lymphocyte apoptosis may play an important role in the pathogenesis of HNL.^9^

The incidence of pleural effusion in HNL is low and rarely reported clinically, so we cannot reach any conclusion regarding the likelihood that HNL patients might have pleural effusion. In the present case, the patient had an exudate. The mechanism of its occurrence remains unclear, and it is possible that the disease itself may lead to pleural disruption, thus causing exudative pleurisy; however, there is a lack of histological evidence to support this concept. Pleural biopsies can easily miss the location of the lesion, since they can only collect a relatively small amount of the sample, and they may be prone to false negative results. On the other hand, thoracoscopy or thoracotomy examinations are highly invasive and are not very popular with most patients. Additionally, the possibility that HNL might be caused by an endogenous sex hormone dysfunction cannot be ruled out, since corticosteroid therapy has been observed to lead to complete reabsorption of pleural effusion. The mechanism of pleural effusion in HNL patients still need to be further investigated.

We searched for similar cases in different databases (PubMed, Embase and LILACS) using the following terms: “histiocytic necrotizing lymphadenitis” AND “pleural effusion” or “Kikuchi-Fujimoto disease” AND “pleural effusion” (Table 1). We found that only one case had been published.^10^

**Table 1.** Results from a search of the literature in medical databases, for case reports on “histiocytic necrotizing lymphadenitis with bilateral pleural effusion diagnosed via cervical lymph node biopsy”. The search was conducted on December 22, 2016

Database	Search strategies	Papers found	Related papers
MEDLINE (via PubMed)	((histiocytic necrotizing lymphadenitis[Title]) AND pleural effusion[Title])	0	0
MEDLINE (via PubMed)	((Kikuchi-Fujimoto disease[Title]) AND pleural effusion[Title])	1	1
Embase (via Elsevier)	((histiocytic necrotizing lymphadenitis[Title]) AND pleural effusion[Title])	0	0
LILACS (via BVS)	((histiocytic necrotizing lymphadenitis[Title]) AND pleural effusion[Title])	0	0

The treatments for HNL include non-steroidal anti-inflammatory drugs and corticosteroids; antibiotic treatment is often ineffective. Although non-steroidal anti-inflammatory drugs can elicit partial effects, there have been reports suggesting that this treatment may cause patients to be more prone to relapse.^11^ It has been suggested that early glucocorticoid therapy should be used to treat HNL, and that the duration and intensity of the treatment should be based on the patient’s response to hormone therapy and the results from follow-up examinations.^12^

## CONCLUSIONS

Pleural effusion is a very uncommon manifestation of HNL that has rarely been reported in these patients. We presented a case of HNL with bilateral pleural effusion that was diagnosed using cervical lymph node biopsy and which was successfully treated with prednisone.

HNL patients are often admitted to a respiratory department, and it is easy to misdiagnose them as having tuberculous pleurisy or other diseases. We reported on this patient in order to improve clinicians’ understanding of such diseases and help reduce the likelihood of misdiagnosis.
